# Task-dependency and structure-dependency in number interference effects in sentence comprehension

**DOI:** 10.3389/fpsyg.2015.00349

**Published:** 2015-04-10

**Authors:** Julie Franck, Saveria Colonna, Luigi Rizzi

**Affiliations:** ^1^Laboratoire de Psycholinguistique, University of GenevaGeneva, Switzerland; ^2^Centre National de la Recherche Scientifique – University of Paris 8Paris, France; ^3^Department of Linguistics, University of GenevaGeneva, Switzerland; ^4^Interdepartmental Centre for Cognitive Studies of Language, University of SienaSiena, Italy

**Keywords:** number, agreement, attraction, intervention, intermediate traces, c-command, cue-based retrieval, comprehension

## Abstract

We report three experiments on French that explore number mismatch effects in intervention configurations in the comprehension of object A’-dependencies, relative clauses and questions. The study capitalizes on the finding of object attraction in sentence production, in which speakers sometimes erroneously produce a verb that agrees in number with a plural object in object relative clauses. Evidence points to the role of three critical constructs from formal syntax: intervention, intermediate traces and c-command (Franck et al., 2010). Experiment 1, using a self-paced reading procedure on these grammatical structures with an agreement error on the verb, shows an enhancing effect of number mismatch in intervention configurations, with faster reading times with plural (mismatching) objects. Experiment 2, using an on-line grammaticality judgment task on the ungrammatical versions of these structures, shows an interference effect in the form of attraction, with slower response times with plural objects. Experiment 3 with a similar grammaticality judgment task shows stronger attraction from c-commanding than from preceding interveners. Overall, the data suggest that syntactic computations in performance refer to the same syntactic representations in production and comprehension, but that different tasks tap into different processes involved in parsing: whereas performance in self-paced reading reflects the intervention of the subject in the process of building an object A’-dependency, performance in grammaticality judgment reflects intervention of the object on the computation of the subject-verb agreement dependency. The latter shows the hallmarks of structure-dependent attraction effects in sentence production, in particular, a sensitivity to specific characteristics of hierarchical representations.

## Introduction

The wide literature on agreement in sentence production has given rise to a large body of research on the phenomenon of interference called ‘attraction.’ In the standard and most explored case, the speaker incorrectly produces a verb that agrees with a plural noun situated in a modifying prepositional phrase (PP) linearly intervening between the subject and the verb (e.g., ^∗^*The time for fun and games are over*, from Bock and Miller, 1991; Bock and Cutting, 1992). As experimental evidence accumulated, it has become evident that various types of syntactic elements have the potential to trigger interference, including adjuncts (Franck et al., 2004) and immediately preverbal objects (Fayol et al., 1994; Hartsuiker et al., 2001; Hemforth and Konieczny, 2003; Konieczny et al., 2004; Franck et al., 2006, 2010; Häussler, unpublished), but also and more intriguingly elements that are not situated between the subject and the inflected verb in the linear word string. Such cases of interference have been reported in object relative clauses as illustrated in (1a) (Bock and Miller, 1991; Franck et al., 2006, 2010), questions (Vigliocco and Nicol, 1998), as well as cleft sentences (Franck et al., 2006). The present study addresses the question of whether similar interference effects are detectable in sentence comprehension. In particular, the work aims to address three questions. First, do the critical constructs from formal syntax, i.e., intervention, intermediate traces, and c-command, which capture attraction patterns in agreement production (Franck et al., 2006, 2010), also play a role in the computation of agreement in sentence comprehension? Second, are the processes involved in the computation of agreement features the same in production and comprehension? Third do different experimental techniques in comprehension tap on distinct aspects of agreement computation in performance?

(1) a. Jean parle aux **patientes** [_RC_ que le médicament ^∗^guérissent.]
           Jean speaks to-the-PL patients-PL that the medicine-SG ^∗^cure-PL.
          ‘John speaks to the patients whom the medicine ^∗^cure.’

     b. Jean dit aux **patientes** [_CC_ que le médicament guérit.] *Jean tells to-the-PL patients-PL that the medicine-SG cures-SG.*
         ‘John tells the patients that the medicine cures.’

The paper is structured as follows. We first present the syntactic configurations underlying object interference. We then turn to the role of the experimental task in agreement computation. Subsequently, we report three experiments exploring the role of syntactic configurations in object interference in sentence comprehension. The first two experiments use different methodologies to test the role of movement and intermediate traces: they contrast attraction in the minimal structural pair consisting of object relative clauses, involving movement (as in 1a), and the superficially similar complement clauses without movement (as in 1b). The third experiment tests the role of c-command by manipulating attraction from moved complex objects. These objects involve both a c-commanding DP and a purely preceding DP within a PP modifier whose respective effects on attraction are systematically assessed (e.g., Quelles **patientes** du médecin dis-tu que le juriste défend? *Which-PL patients-PL of the doctor do you say that the-SG lawyer-SG defends-SG?* vs. Le chirurgien de quelles **patientes** dis-tu que le juriste défend? *The surgeon of which-PL patients-PL do you say that the-SG lawyer-SG defends-SG?*).

### The Role of Syntactic Structure in Object Interference

In a detailed exploration of object attraction in sentence production, Franck et al. (2010) tested various hypotheses with respect to the structural conditions underlying agreement errors. The starting point of their work was the finding that despite its close superficial resemblance to the object relative (1a), the sentence complement clause (1b) fails to trigger attraction. Whereas in (1a) *patients* is the moved object of the target verb *cure* used transitively, in (1b) it is the unmoved object of the main verb *tells* while the target verb is used intransitively.

In four additional experiments, the authors explored the role of properties that distinguish relative clauses from complement clauses. Argumenthood was found to play no role in attraction since objects that are not part of the argument structure of the target verb, as in extraction from clausal complements, trigger similar attraction as thematic objects (e.g., Voici **les otages** que le journaliste ^∗^apprennent qu’on a blessés; *Here are the hostages-PL that the journalist-SG ^∗^learn-PL that someone injured*), while objects in their canonical post-verbal position did not generate attraction either. Participle agreement triggered by the object in French was also found to play no role in attraction, since attraction effects were found with elements that fail to trigger participle agreement like accusative clitics in the causative construction (e.g., Le directeur **les** ^∗^font acheter; *The director-SG them-PL make-PL ^∗^buy*). Moreover, the strength of object attraction in structures in which the object has moved to the front of the sentence and fails to intervene linearly between the subject and the verb (relatives and clefts) was shown to be of a similar strength to that of a linearly intervening object, as is the case of the clitic object pronoun (e.g., L’avocat **les** ^∗^défendent; *The-SG lawyer them-PL* ^∗^*defend*, Franck et al., 2006). All these cases involve an object (or its trace) intervening in a c-commanding position between the terms of the agreement relation, the subject and the agreeing verb (see Franck et al., 2006, 2010 for a graphical illustration of the hierarchical structure and c-command relations). Evidence suggests that attraction is significantly weaker if the attractor intervenes purely in terms of precedence, i.e., in a position situated lower down in the tree and that fails to c-command the agreement node, as is the case of the modifying PP (e.g., L’avocat des **patientes** ^∗^mentent; *The-SG lawyer of the patients-PL* ^∗^*lie-PL*) or the dative clitic (e.g., L’avocat **leur** ^∗^mentent; *The-SG lawyer to them-PL* ^∗^*lie, The lawyer lie to them*, Franck et al., 2006). In sum, the empirical evidence suggests that object movement is a necessary and sufficient condition for object attraction to arise in an SVO language like French (conditions may differ for SOV languages if the object originates in preverbal position), and that c-commanding attractors generate more interference than preceding ones.

But why does the object interfere with agreement even in cases like (1a), in which it is pronounced in a position from which it does not intervene, either linearly or hierarchically, between the subject and the verb? Franck et al. (2006, 2010) noted that interference with the object seems to occur at a position that is neither the final nor the initial position since these two positions failed to generate attraction. The authors proposed that the intermediate position of the object in the hierarchical structure, mediating its initial position in the thematic structure and its final surface position, plays a crucial role. In a (much simplified) representation of example (1a) like, *Jean parle aux* ***patientes*** *[_RC_ que le médicament* ***t_**2**_****^∗^guérissent* ***t_**1**_****]*, the object *patientes* is initially generated in **t_**1**_** and then moves in **t_**2**_**, a position which intervenes on the subject-verb agreement (AGREE) relation between the position hosting agreement morphology (ultimately attached to the verb) and the subject in its initial thematic position. Finally, the object moves higher to reach its final position. The intermediate position **t_**2**_**, unpronounced but with morphological reflexes in some cases such as participial agreement in French (Kayne, 1989) or wh-agreement in Austronesian languages (Chung, 1998), is postulated in formal syntactic analyses for locality reasons (e.g., to respect Phase Impenetrability in a system like Chomsky, 2001; see Gibson and Warren, 2004 for experimental evidence for the role of intermediate traces in the processing of long-distance dependencies). So, we argued that formal characteristics of abstract representations assumed in the “principles and parameters”/minimalist analysis of agreement form the representational basis over which agreement processes operate in performance. The intermediate trace of the object in the vP periphery may be thought of, in a phase-based architecture, as corresponding to a temporary memory buffer from which the object remains active and available for further processes (Chesi, 2005). The activation of the object in memory in this precise position intervening on agreement would be the locus of the interference effects observed in agreement production.

### Task-Effects in Object Interference

Production and comprehension critically differ in that whereas in production the speaker has access to the conceptual structure of the sentence, this structure is incrementally built in sentence comprehension, under the strong guidance of predictive mechanisms (see e.g., Hale, 2001; Levy, 2008). Nevertheless, under the view that the effects reported in agreement production reflect properties of the hierarchical structure over which agreement is calculated, one expects the same effects to show up in sentence comprehension (e.g., see Kempen et al., 2012, for a model with shared representations and shared processes of syntactic encoding and decoding). Various studies have shown that plural attractors situated in the subject phrase interfere with verb agreement processing in sentence comprehension (e.g., Nicol et al., 1997; Pearlmutter et al., 1999; Pearlmutter, 2000; Kaan, 2002; Thornton and MacDonald, 2003; Häussler, unpublished). The typical finding is that participants spend more time reading or judging the acceptability of a sentence in the presence of a plural mismatching subject modifier as compared to when it is singular.

However, only a few studies have explored object attraction in comprehension. Clifton and colleagues conducted two grammaticality judgment studies exploring attraction in structures like (2) (Clifton et al., 1999). They found that although participants correctly reject ungrammatical sentences like (2a), they tend to accept ungrammatical sentences like (2b) in which a plural element (people) is situated higher than the subject and the verb. Clifton et al. argued that the relative acceptability of (2b) lies in the fact that the chain between the moved element and its trace is still active, and the agreement dependency is on its path. The effect was attributed to the passing of the plural feature on the agreement dependency and not to a late gap complexity effect affecting the general difficulty in processing (2b), since the same sentences with a singular attractor (*people* was replaced by *person*) were systematically rejected, attesting that the difficulty selectively arises in the presence of the plural feature (see also Kayne, 2000 for the hypothesis that the agreement pattern in (2b) is fully grammatical in some dialects). The authors concluded that interference arises because of the link of the projection path that is shared by two distinct syntactic dependencies (agreement and the NP-trace chain). These findings and the overall interpretation are consistent with the analysis (just summarized) in Franck et al. (2006, 2010), which would further specify that interference would be triggered in (2b) by the transit of the plural relative head *people* in the periphery of the vP headed by *think*, a position from which *people* hierarchically intervenes between *manager* and the agreeing head, thus giving rise to the plural form *think.*

(2) a. Lucine dislikes **the people** who think the manager ^∗^know the answers.
      b. Lucine dislikes **the people** who the manager (^∗^)think **t** know the answers.

Using a methodology based on a two-choice response time paradigm, Staub (2009, 2010) also found evidence for interference from moved objects when participants were asked to select between the two verb forms (singular vs. plural) presented simultaneously on the screen after being exposed to the subject phrase one word at a time in a Rapid Serial Visual Presentation (RSVP) procedure. Verb selection was found to be sensitive to attraction similarly to sentence production, with slower response times to select the correct verb form in the presence of a mismatching plural feature on the object of the sentence. Note, however, that it is unclear whether the task taps into comprehension or production, since both components are involved.

Wagers et al. (2009) investigated object interference in two experiments on object relative clauses (Experiments 2 and 3) involving a self-paced reading procedure. The results showed a significant effect of the object number in the region following the critical verb. However, the effect was restricted to ungrammatical sentences and showed up in the form of faster reading times in the presence of a mismatching plural attractor, suggesting that it reflects a grammatical illusion lying in the incorrect computation of agreement. More generally, across the five self-paced reading experiments on both object and subject modifier attraction, the authors consistently found no attraction in grammatical sentences. This finding appears prima facie to be in contradiction with the other reports of significant interference in grammatical sentences involving subject modifiers, but the careful testing of these structures by Wagers et al. (2009) suggests that the attraction effect observed at the verb in these studies was actually a spillover effect from the plural feature preceding the verb. In line with that interpretation, they found that the introduction of an adverb between the modifier and the verb dissolved the effect (e.g., The slogan on the posters unsurprisingly was designed to get attention). Recent evidence from on-line experimental techniques further supports the view that attraction is restricted to ungrammatical sentences (eye-tracking: Dillon et al., 2013; ERP: Tanner et al., 2014). The possibility that attraction only arises in ungrammatical sentences in comprehension has important consequences for models of agreement computation. Wagers et al. (2009) suggest that attraction in sentence comprehension is driven by the properties of a cue-based retrieval process triggered when the parser encounters an agreement error: the system involves a predictive component by which the parser expects a particular number feature on the verb, and only if the bottom-up features of the verb mismatch the top–down predicted features is the cue-based-retrieval deployed to check whether the correct feature was missed during the first pass. On this view, number interference in comprehension arises from a fundamentally different cause from attraction errors in sentence production.

Nevertheless, as Wagers et al. (2009) point out, other studies reported reliable interference effects in grammatical sentences that cannot easily be explained by an extended effect of the plural attractor. In a series of five experiments using a Maze task (requiring for each upcoming word in the sentence to select amongst two words, and in the critical region, between a correctly agreeing verb and a word from a different grammatical category, e.g., *was* vs. *ink*) or a sentence classification task (requiring to determine whether the sentence is a legitimately ordered string of words), Nicol et al. (1997) found significant interference in grammatical sentences. Since in these tasks response times reflect either the selection of the correct grammatical category or the global assessment of the sentence word order, the interference found does not seem to be attributable to the spillover of the plural feature processing on the verb. In a speeded grammaticality judgment task on sentences containing an embedded clause with complex subjects modified by a genitive phrase, Häussler et al. (2003) reported interference from the plural attractor in grammatical sentences only; no interference was found in ungrammatical sentences. Using a similar procedure of grammaticality judgment, Häussler and Bader (2009) also found interference from a mismatching feature within a relative clause introduced by a possessive pronoun in both grammatical and ungrammatical sentences. Here again, the slow down observed in mismatch condition cannot be attributed to a spillover effect of processing the plural feature on the attractor linearly preceding the verb.

Summing up, while some studies of attraction in sentence comprehension point to similarities with sentence production, others suggest differences. Moreover, discrepancies are also found between studies of attraction in sentence comprehension, some reporting attraction in ungrammatical sentences only, others in both grammatical and ungrammatical sentences, and yet others finding attraction in grammatical sentences only. However, a direct comparison across these studies is difficult due to the fact that they involve different tasks and different syntactic structures. The role of the task in language performance gained increased interest in the recent years (e.g., Caplan et al., 2008; Caplan, 2010; see also Salverda et al., 2010 for an overview of task effects in the visual world paradigm), and it therefore seems crucial to collect finely controlled comparisons on agreement performance before conclusions be drawn with respect to the mechanism of attraction.

In order to test the potential influence of the task on attraction, Experiments 1 and 2 use the same materials tested in agreement production by Franck et al. (2010), but with two different experimental methods. Experiment 1 uses a similar self-paced reading procedure to that used by Wagers et al. (2009) but differs from it in that only grammatical sentences were introduced, to maximize the naturalness of the comprehension process and avoid any potential contamination from the presence of ungrammatical sentences. Experiment 2 uses a procedure of speeded grammaticality judgment on the ungrammatical versions of these sentences, with the aim of maximally promoting agreement computation in comprehension. Finally, since Experiment 2 only involved ungrammatical sentences, Experiment 3 manipulated grammaticality in order to assess its role in the same procedure of speeded grammaticality judgment used in Experiment 2.

## Experiment 1: Object Interference in Self-Paced Reading

Experiment 1 explores the role of object movement, modulated by its number specification, in a maximally natural sentence comprehension task involving the self-paced reading of grammatical sentences. The same materials as in Experiment 1 from Franck et al. (2010) were used, involving a minimal contrast between a structure with object movement in an object relative clause (1a), and a structure without object movement in a complement clause (1b). The two structures are identical in surface order; the main difference lies in the selection of the main verb, which takes a single complement in (1a) (thus enforcing the analysis of the *que* clause as a relative modifying the object DP) and two complements in (1b) (thus giving rise to a sentential complement interpretation for the *que* clause). Interference is examined on the agreement of the verb in the subordinate clause (to cure). In the relative clause (1a), ‘patient(s)’ is the object of the target verb ‘cures,’ and is therefore assumed to transit via the intermediate position at the embedded vP periphery intervening on the subject-verb agreement relation (Franck et al., 2010). In the complement clause (1b), ‘patient(s)’ is the unmoved indirect object of the main verb while the embedded verb ‘cures’ is used intransitively. If the intervention of the intermediate trace of the moved object on agreement creates interference in sentence comprehension similarly to sentence production, a slow down is expected at the verb in the presence of a mismatching plural object as compared to a singular object in object relatives (1a), but not in sentence complements (1b). However, if attraction in sentence comprehension reflects a process of ‘rechecking’ triggered by an erroneous agreement, no interference is expected from the plural feature in either of the two structures.

### Method

#### Participants

Seventy-two students from the University of Geneva, aged between 18 and 40, took part in the experiment. They received course credit for their participation. The experiment was approved by the ethics committee of the Department of Psychology of the University of Geneva and informed consent was obtained from all participants.

#### Materials

The experimental materials consisted of the 24 sentences used in Franck et al. (2010) incorporated in a 2 × 2 design crossing structure (relative vs. complement) and the number of the object (singular vs. plural). All subject head nouns were singular. Subjects and objects were all animate. Since the initial sentences ended with the target verb, two windows were added after the verb in order to measure potential spillover effects. These windows contained an adverb followed by a locative phrase. Each sentence was decomposed into 8 windows corresponding to phrases (content word + grammatical word if present). All test sentences were grammatical with respect to subject-verb agreement. Each sentence was followed by a yes/no comprehension question that probed participants’ interpretation of the thematic relations in the sentence. Examples of test items are presented in **Table 1** (the full list of items is available in the Supplementary Materials).

**Table 1:** Example of item in the four experimental conditions of Experiment 1.

Relative clause	Match	Jérôme/parle/à la prisonnière/que/le gardien/sort/parfois/dans la cour. *Jérôme/speaks/to the prisoner-SG/that/the guard-SG/takes-SG out/sometimes/in the yard.*
	Mismatch	Jérôme/parle/aux prisonnières/que/le gardien/sort/parfois/dans la cour. *Jérôme/speaks/to the prisoners-PL/that/the guard-SG/takes-SG out/sometimes/in the yard.*

Sentence complement	Match	Jérôme/dit/à la prisonnière/que/le gardien/sort/parfois / dans la cour. *Jérôme/tells/the prisoner-SG/that/the guard-SG/goes-SG out/sometimes/in the yard.*
	Mismatch	Jérôme/dit/aux prisonnières/que/le gardien/sort/parfois/dans la cour. *Jérôme/tells/the prisoners-PL/that/the guard-SG/goes-SG out/sometimes/in the yard.*

An additional set of 48 grammatical filler items were built. Half of them had the same structure as the experimental materials (12 Object relative clauses and 12 Complement clauses) but with plural subjects (half with singular objects). The other half involved a variety of syntactic structures (eight declaratives, eight relatives, four temporal modifiers, four PP modifiers) with a varying number of reading windows.

#### Procedure

Sentences were presented on a computer screen using the e-prime software in a self-paced moving window paradigm (Just et al., 1982). Each sentence was first presented with dashes replacing words. Participants were instructed to read sentences by pressing the space bar in order to have the segments appear. Once read, windows disappeared from the screen such that only one window was readable at a time. Participants were told that they would also have to answer yes/no comprehension questions about the content of these sentences. Instructions encouraged both rapid reading and correctness in answering the questions. Order of presentation of the sentences was randomized. The experiment started with four practice trials.

#### Data analyses

Analyses of reading times were run after excluding incorrect responses to comprehension questions (181 data points rejected representing 10.2% of the data). Remaining response times were then trimmed for outliers, defined as data points with a value above 3 s for all participants and all regions (representing 3.1% over all responses). They were treated as missing values. Log-transformed response times and accuracy proportions were analyzed with (generalized) linear mixed-effects regression models with random intercepts for participants and items (Baayen et al., 2008), using the statistical software R (R Development Core Team, 2013). Estimates, *t*-values (for LME), *z*-values (for GLME) and *p*-values for the fixed factors and interactions were obtained via the lmerTest package, which provides *p*-values calculated based on Satterthwaite’s approximation. Significant interactions were further explored with (generalized) linear mixed-effects regression models separately on each of the two modalities of one of the variables involved in the interaction.

### Results

#### Reading Times

**FIGURE 1:**
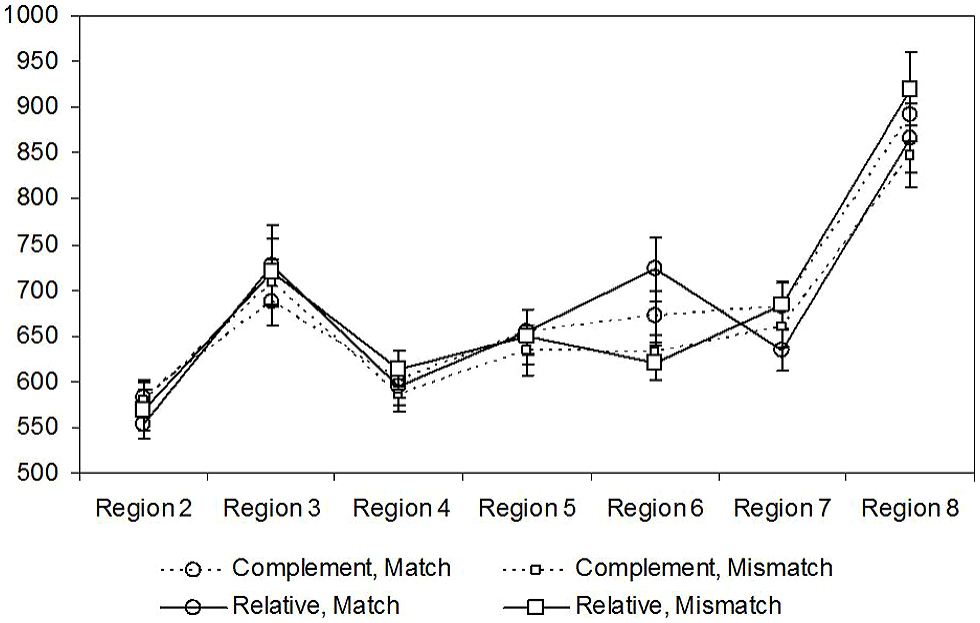
Distribution of reading times (in ms) in the four experimental conditions of the different regions of Experiment 1. The critical region containing the test verb is Region 6.

The distribution of reading times across the different experimental conditions in the different regions is reported in **Figure 1**.

*Region 2 (main verb).* A marginal effect of structure was found with slower response times on the main verb of the relative clause condition (581 ms) than on the main verb of the complement clause (561 ms; β = -0.033, *t* = -1.76, *p* = 0.081). There was no effect of number and no interaction (*ts* < 1).

*Region 3 (object NP).* No main effect or interaction was significant (all *ts* < 1).

*Region 4 (complementizer)*. No main effect or interaction was significant (all *ts* < 1).

*Region 5 (subject NP).* No main effect or interaction was significant (all *ts* < 1).

*Region 6 (target verb).* A significant effect of number was found, with slower response times for singular objects (695 ms) than for plural objects (623 ms; β = -0.078, *t* = -2.73, *p* = 0.006). The main effect of structure was not significant (*t* < 1), but it entered into an interaction with number (β = -0.013, *t* = -1.94, *p* = 0.053). Subsequent models exploring the interaction showed that whereas number significantly affected reading times in the relative clause condition (β = -0.115, *t* = -2.23, *p* = 0.027), it failed to affect them in the complement clause condition (*t* < 1).

*Region 7 (adverb).* A trend toward slower response times in the complement clause condition (671 ms) than in the relative clause condition (657 ms) was found (β = -0.029, *t* = -1.34, *p* = 0.180). There was no effect of number and no interaction (*ts* < 1).

*Region 8 (locative).* A trend toward an interaction between number and structure was found (β = 0.055, *t* = 1.17, *p* = 0.24). There was no effect of number or structure (*ts* < 1).

#### Accuracy

**FIGURE 2:**
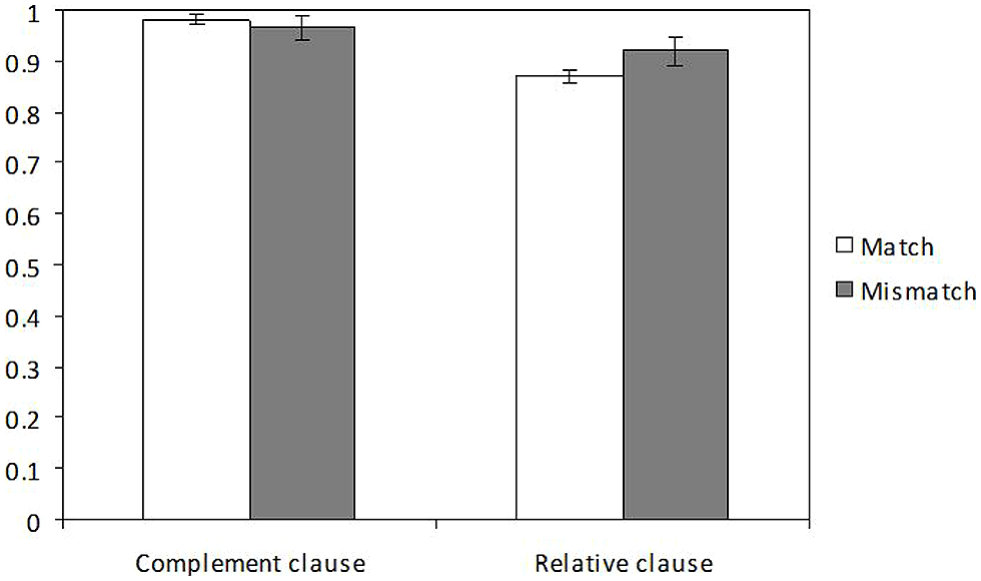
Accuracy proportions in the comprehension questions of Experiment 1

The distribution of mean accuracy scores in the four experimental conditions is illustrated in **Figure 2**. Generalized linear mixed effect analysis showed that accuracy was significantly higher in the complement clause condition (0.97) than in the relative clause condition (0.89; *p* < 0.001; β = -1.467, *z* = -3.89, *p* < 0.001). The interaction and the number effect were not significant (*ts* < 1).

### Discussion

Experiment 1 shows that the object’s number feature influences processing speed at the verb in the object relative clause condition, but not in the corresponding complement clause condition, despite the surface similarity between the two structures. The observed effect shows that a feature mismatch between the extracted object and the subject of the relative clause speeds up reading of the verb segment. As mentioned in the Introduction, the experimentally elicited production of agreement is made more error-prone by the presence of a feature mismatch. Under the hypothesis that number mismatch influences the computation of agreement similarly in production and comprehension, one would have expected to find that it slows down sentence comprehension processes. Rather, it appears that a feature mismatch makes production of the relative clause verb error-prone, but reading of the relative clause verb faster. Why does a featural mismatch trigger opposite results in production and comprehension? Three observations suggest an answer, which is that the comprehension task did not tap into the mechanism of agreement computation, but rather in a mechanism of chain resolution responsible for linking a moved element to its trace.

First, the present experiment shows an influence of the object’s number in grammatical object relative clauses, which contrasts with the study by Wagers et al. (2009) who only found an effect in object relatives that contained an agreement error. Wagers et al. (2009) suggested that the interference effect they reported reflects reanalysis: only if the verb feature conflicts with the predicted feature would a cue-based retrieval process be deployed to actively retrieve the matching feature in the parsed tree. Our finding of object interference in the context of naturally reading a grammatical sentence cannot be accounted for by this view that the mismatch effect only arises as part of a second-pass process of agreement ‘rechecking.’

Second, our finding that participants were faster in the presence of a plural feature mismatching the singular subject than in the presence of a singular feature matching the singular subject also contrasts with various comprehension studies showing a detrimental effect associated with a plural mismatching feature, whether it is on a subject modifier or a moved object, and whether comprehension is tested by way of self-paced reading or more indirect experimental procedures like maze tasks, classification tasks, grammaticality judgment or two-choice verb selection (Nicol et al., 1997; Clifton et al., 1999; Pearlmutter et al., 1999; Pearlmutter, 2000; Staub, 2009, 2010; Häussler, unpublished).

Third, although the direction of the interference effect in Experiment 1 may, at first glance, appear to be in line with that reported by Wagers et al. (2009) who also found faster reading times with plural mismatching objects, the two effects fundamentally differ since we found the effect in grammatical sentences whereas Wagers and colleagues found it in ungrammatical sentences. In the latter case, the parser is lured by the presence of a plural feature on the object creating an illusion of grammaticality. Hence, the finding that participants were faster in the plural mismatch condition also argues against a structure-based feature spreading mechanism like the one assumed to take place in production (Nicol et al., 1997; Vigliocco and Nicol, 1998; Franck et al., 2002; Eberhard et al., 2005). This raises the intriguing possibility that a different mechanism underlies the mismatch effect found here.

How do these different aspects of the data inform us about the mechanism underlying the number effect found in the present experiment? Directly relevant to the present study are the recent reports in the acquisition literature of intervention effects in the comprehension of object relatives. Adani et al. (2010) and Adani (unpublished) found that both English and Italian speaking children showed better performance in a sentence-picture matching task when the object and the subject of the object relative clause mismatched in number (e.g., *Show me the elephant that the lions are washing* is better understood than *Show me the lion that the elephant is washing*). Using a similar task Belletti et al. (2012) reported better performance in Hebrew-speaking children for sentences involving a gender mismatch between the subject and the object relative. Empirical evidence suggests that only features that play an active role as triggers of syntactic movement have the potential to influence comprehension. This conclusion was reached on the basis of cross-linguistic evidence showing that in contrast to Hebrew children, Italian children failed to show a comparable sensitivity to gender mismatch in their comprehension of object relative clauses: this property was related to the different syntactic status of gender agreement in Italian.

According to the version of Relativized Minimality (Rizzi, 1990, 2004) assumed in the references quoted (along the lines developed in Friedmann et al., 2009), what makes the relevant kind of object relatives problematic for children is the intervention of the subject DP in the path connecting the relative head and its trace in object position. In particular, the difficulty is attributed to the set-theoretic relation of inclusion that characterizes the feature make-up of the object and that of the subject. When both the object and the subject are singular, the object is endowed with features [+R, +N, +Sg] (where +R is the feature designating the relative head) whereas the subject is endowed with features [+N, +Sg]: hence, the featural make-up of the intervener is included in the one of the antecedent. Friedmann et al. (2009) proposed that inclusion is problematic for children to explain their difficulty with making the required connection between the object and its trace. However, if the subject is plural, the number mismatch creates an intersection set, the object carrying [+R, +N, +Sg] and the subject [+N, +Pl]. Intersection is higher than inclusion in a natural scale of distinctness, a relation that is assumed to be accessible to the child’s system in Belletti et al. (2012). Transposing this approach to the adult data collected in Experiment 1, the slowing down of reading time at the verb in the match condition as compared to the mismatch condition may be interpreted as an indication of the same gradation observed in children, inclusion being more difficult than intersection.

In this view, there is no contradiction between the number effect found in the production and comprehension studies of object relatives: whereas the production experiments directly tap into the agreement process, requiring the choice of a properly agreeing form, an operation which is penalized by the presence of a mismatching intervener in the immediate vicinity, the reading of the sentences primarily reflects the process of structure building, and in particular of the building of an appropriate A’-chain across an intervener, an operation which is enhanced by number mismatch. Hence the seemingly opposite consequences of mismatch in production and comprehension may be seen as a byproduct of the specific demands of the experimental tasks. If self-paced reading, as it is used in Experiment 1, mostly reflects the time taken by the parser to build the sentence structure and resolve the A’-dependency, it does not directly bear on agreement computation; one direct prediction of that account would be that the same effect of feature mismatch should be observed in sentences that do not involve an agreement configuration in their structure. We are currently exploring that possibility.

If self-paced reading does not tap into agreement processes, at least when complex sentences involving movement are involved, it may be relevant to identify a task that taps into the component of agreement processing in sentence comprehension. Experiment 2 uses a speeded grammaticality judgment task with sentences involving ungrammatical agreement, such that participants were forced to process agreement. If the same computational principles of agreement are at play in this task as in production, Experiment 2 should uncover the same structure-dependent attraction effects as found in sentence production.

## Experiment 2: Object Attraction in Speeded Grammaticality Judgment

Experiment 2 tests the same experimental conditions as Experiment 1, contrasting object relatives and sentence complements, but this time with a speeded grammaticality judgment task in which agreement computation is explicitly assessed. Hence, in this task, agreement markers cannot be used for the structure building process; rather, agreement can only be computed once the hierarchical structure has been built. If the grammaticality judgment task allows tapping specifically into agreement processing in sentence comprehension, and if this process shows the signature of intervention effects as reported in sentence production, interference is expected to show up selectively in the condition where the object intervenes on the agreement dependency, i.e., in object relatives. In contrast, no interference is expected from the object of the main verb in sentence complements. Moreover, interference should take the shape of an attraction effect, with slower judgment times in the presence of plural mismatching objects.

### Method

#### Participants

Thirty students of the University of Geneva, different from Experiment 1 and aged between 18 and 40, took part in the Experiment. They received credits for their participation. The experiment was approved by the ethics committee of the Department of Psychology of the University of Geneva, and informed consent was obtained from all participants.

#### Materials

Materials consisted of the same 24 test items as Experiment 1 without the last two windows, such that all sentences ended with the target verb. All sentences were ungrammatical with respect to subject-verb agreement: the verb in the subordinate clause was plural with a singular subject head noun. In addition to the test items, 120 filler items were built. Forty-eight of them were of the same structure as the experimental items; 16 correct with a singular subject (half with a singular object), 16 correct with a plural subject (half with a singular object) and 16 incorrect with a plural subject (half with a singular object). The 72 remaining items had a different structure, with a subject modifier intervening linearly between the subject and the verb. Half of the modifiers consisted of subject relative clauses (e.g., Jean parle au gardien des bâtiments qui dort), the other half consisted of complement clauses (e.g., Jean dit que le programme des expériences fonctionne). Thirty-six (half) of these sentences were correct, the other half were incorrect. Half had a singular subject, the other half had a plural subject. Examples of test items are presented in **Table 2**.

**Table 2:** Example of an item in the four experimental conditions of Experiment 2.

Relative clause	Match	^∗^ Jérôme parle à la prisonnière que le gardien sortent. *^∗^ Jérôme speaks to the prisoner-SG that the guard-SG take out-PL.*
	Mismatch	^∗^Jérôme parle aux prisonnières que le gardien sortent. *^∗^ Jérôme speaks to the prisoners-PL that the guard-SG take out-PL.*

Sentence complement	Match	^∗^ Jérôme dit à la prisonnière que le gardien sortent. *^∗^ Jérôme tells the prisoner-SG that the guard-SG go out-PL.*
	Mismatch	^∗^ Jérôme dit aux prisonnières que le gardien sortent. *^∗^ Jérôme tells the prisoners-PL that the guard-SG go out-PL.*

#### Procedure

Materials were presented on a computer screen using the e-prime software. Sentences were split in windows corresponding to phrases (content word + grammatical word if present). Windows were presented for a fixed period of 500 ms, except at the verb, i.e., the final word of the sentence. These rather long presentation times were selected in order to minimize judgment errors, and avoid a possible trade-off between speed and accuracy. Grammaticality judgment times were measured at the verb onset. Participants were asked to judge the grammaticality of the sentences as quickly as possible and press on the corresponding response button. Pressing the button made the next window appear, such that a sustained rhythm was imposed.

#### Data Analyses

Incorrect grammaticality judgments representing 7.8% of the data were removed from the response times analyses. Analyses of response times were run both on the full dataset as well as on the data trimmed for outliers, defined as responses slower than 3 s (representing 7.9% of the data). Since both models provided similar outputs, the model of the complete data set is reported. Log-transformed response times and accuracy proportions were analyzed by way of (generalized) linear mixed-effects regression models with random intercepts for participants and items (Baayen et al., 2008), following the same procedure as for Experiment 1.

### Results

#### Response Times

**FIGURE 3:**
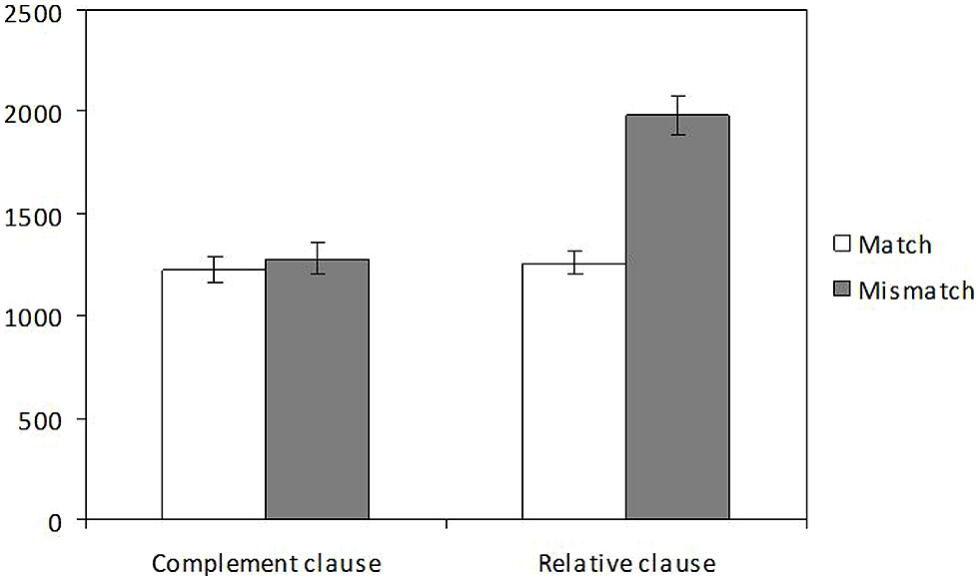
Distribution of RTs (ms) for correct judgments at the target verb in the speeded grammaticality judgment task of Experiment 2

The distribution of response times is illustrated in **Figure 3**. Mixed models revealed a main effect of number, with slower RTs with plural objects (1619 ms) than with singular ones (1242 ms; β = 0.186, *t* = 4.449, *p* < 0.001) as well as a main effect of structure with slower times for judging the grammaticality of object relatives (1609 ms) than for judging complement clauses (1253 ms; β = 0.179, *t* = 4.277, *p* < 0.001). The model showed a significant interaction between structure and number (β = 0.273, *t* = 3.185, *p* = 0.002): whereas number significantly affected response times in the relative clause condition (β = 0.302, *t* = 4.463, *p* < 0.001), no effect of number was found in the complement clause condition (*t* < 1).

#### Accuracy

**FIGURE 4:**
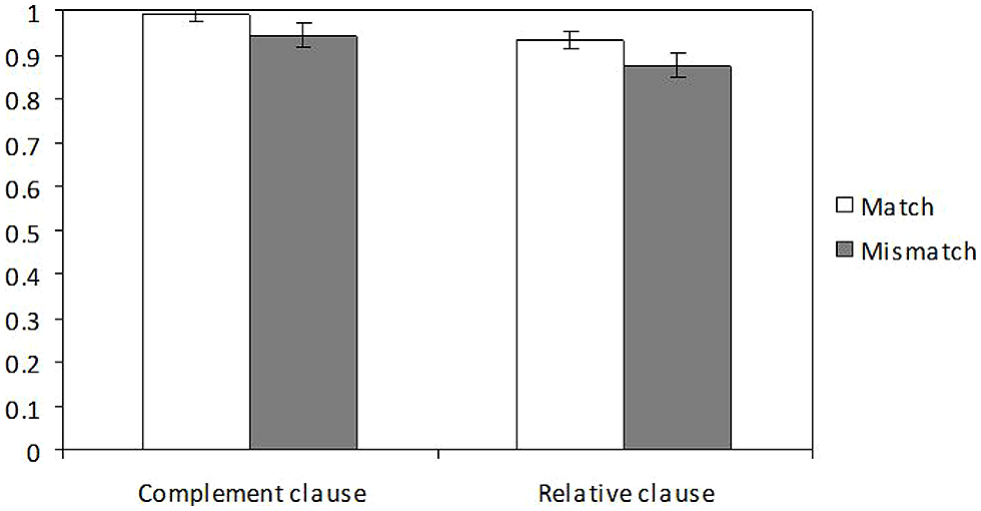
Accuracy proportions in the speeded grammaticality judgment task of Experiment 2

Mean accuracy scores are reported in **Figure 4**. Accuracy was significantly affected by structure with better scores in the complement clause condition (0.97) than in the relative clause condition (0.90; β = -1.379, *z* = -2.18, *p* = 0.03). Number was marginally significant with higher scores for singular objects (0.97) than for plural ones (0.91; β = -1.180, *z* = -1.863, *p* = 0.06). The interaction was not significant (*t* < 1).

### Discussion

In line with the production data reported on the same materials (Franck et al., 2010), participants were disturbed by the presence of a plural object in object relative clauses when performing a grammaticality judgment task bearing on the verbal agreement morphology: they were significantly slower to judge that the sentence was ungrammatical when the object was plural than when it was singular. By contrast, the plural feature in the sentence complement structure generated no or at least significantly reduced interference. The parallelism with production reports finds a natural explanation under the hypothesis that the same mechanism of agreement computation is at play in both tasks. This mechanism is sensitive to the hierarchical intervention of the intermediate trace of the object on the subject-verb dependency, which may have as processing consequence the local reactivation of the object, leading to interference in the processing of agreement, as argued in Franck et al. (2010). Under this hypothesis, the data provide evidence that agreement computation in sentence comprehension operates on the same syntactic representations as in sentence production (e.g., Nicol et al., 1997; Pearlmutter et al., 1999; Thornton and MacDonald, 2003; Hartsuiker, 2006; Badecker and Kuminiak, 2007).

Experiment 2 differs from Experiment 1 in the direction of the number effect: whereas faster response times were observed in the number mismatch condition in the self-paced reading Experiment 1, number mismatch slowed down grammaticality judgments in Experiment 2, in line with attraction effects found in sentence production. One could argue that the opposite direction of the effect found in Experiment 2 as compared to Experiment 1 is due to the fact that whereas Experiment 1 involved grammatical sentences, Experiment 2 involved ungrammatical sentences. Experiment 3 tests whether grammaticality affects the direction of the number interference effect in a grammaticality judgment task. If the effect reported in Experiment 2 merely reflects properties of syntactic representations, grammaticality should not affect performance since the same hierarchical structure underlies grammatical and ungrammatical sentences.

## Experiment 3: The Role of C-Command

Findings in sentence production suggested that c-commanding interveners have a greater potential to trigger interference effects than preceding interveners (Franck et al., 2006, 2010).

Experiment 3 contrasts two conditions involving wh-movement of a complex objects, which potentially interferes with subject-verb agreement while transiting in a vP peripheral position, along the lines illustrated in the Introduction. The property that varies is where, in the complex object, the plural feature is expressed. In (3a) the DP head (hence, the whole object DP) is plural (*quelles patientes du médecin*), while in (3b) (*le chirurgien de quelles patientes*) only the embedded DP within a PP modifier is plural (here the embedded DP pied-pipes the whole object DP triggering its movement to the left periphery).

(3) a. Quelles **patientes** du médecin dis-tu que le juriste défend/^∗^défendent?
          Which-PL patients-PL of the doctor do you say that the lawyer-SG defends-SG/^∗^defend-PL?

     b. Le chirurgien de quelles **patientes** dis-tu que le juriste défend/^∗^défendent?
  *        The surgeon of which-PL patients-PL do you say that the lawyer-SG defends-SG/^∗^defend-PL*?
          Which patients’ surgeon do you say that the lawyer defends?

The crucial point here is that when the complex object DP transits through the vP peripheral position, intervening in the agreement process between the verbal inflection and the subject, the DP with plural marking intervenes in terms of c-command in (3a) (a hierarchical property), while it only intervenes in terms of precedence in (3b), where it is buried within the PP modifier. Under the hypothesis that the same guiding principles operate in sentence production and in the agreement checking process assumed to take place in grammaticality judgment, the plural feature on the c-commanding element ‘patientes’ in (3a) is expected to generate stronger attraction than the plural feature on the preceding element ‘patientes’ in (3b). While Experiment 1 tested grammatical sentences and Experiment 2 tested ungrammatical ones, Experiment 3 manipulates grammaticality in order to provide a systematic assessment of its role on agreement processing in grammaticality judgment.

### Method

#### Participants

Twenty-six students of the University of Geneva different from Experiments 1 and 2 and aged between 18 and 40, took part in the Experiment. They received credits for their participation. The experiment was approved by the ethics committee of the Department of Psychology of the University of Geneva, and informed consent was obtained from all participants.

#### Materials

Materials consisted of 24 sets of 8 items. The variables manipulated include the number of the attractor (singular vs. plural), the position of the attractor with respect to the subject in its base position (c-command vs. precedence), and the grammaticality of subject-verb agreement (grammatical vs. ungrammatical). Questions, rather than declarative object relative clauses, were used to avoid attachment ambiguity (a relative clause could either be attached to the higher DP or to the DP embedded in the modifying PP). The position of the wh-marked element *quelles* was always on the target DP such that it was on the head in the c-commanding condition and on the PP modifier in the precedence condition. In this design, the plural DP was the wh-DP in both the c-command and precedence condition, so that the crucially varying DP would have the same role of wh-operator at Logical Form. As a result, the finding of an effect of structure would attest to a syntactic position effect, not of a semantic/logical form effect. All DPs were animate. An example of item across the eight experimental conditions is presented in **Table 3** (the full list of items is available in the Supplementary Materials).

**Table 3:** Example of an item in the eight experimental conditions of Experiment 3.

C-command	Match	Quelle patiente du médecin dis-tu que le juriste défend/^∗^défendent? *Which patient of the doctor do you say that the lawyer defends/^∗^defend?*
	Mismatch	Quelles patientes du médecin dis-tu que le juriste défend/^∗^défendent? *Which patients of the doctor do you say that the lawyer defends/^∗^defend?*

Precedence	Match	Le chirurgien de quelle patiente dis-tu que le juriste défend/^∗^défendent? The surgeon of which patient do you say that the lawyer defends*/^∗^defend*?
	Mismatch	Le chirurgien de quelles patientes dis-tu que le juriste défend/^∗^défendent? The surgeon of which patients do you say that the lawyer defends*/^∗^defend*?

Thirty-two filler grammatical items with the same structure as the test items but with plural subjects were created. An additional set of 40 fillers (half with singular subjects, half grammatical) was added. These consisted of object relatives (16), subject relatives (16) and PP modifiers in simple structures (8). Items were spread across four experimental lists that each contained 48 test items and the 72 fillers. Each list contained both the grammatical and the ungrammatical version of a test item, presented in two separate blocks with a short pause in between. Each block contained the same number of items in the eight conditions, presented in random order.

#### Procedure and Data Analyses

The same procedure and data analyses as Experiment 2 were adopted. Incorrect grammaticality judgments representing 17.3% of the data were removed from the response times analyses. Again, since the models with and without data trimming were identical, the models reported are those without data trimming. The number of incorrect judgments (216 data points) is small and their distribution is too complex to allow conclusions, nevertheless, the analysis is available in the Supplementary Materials.

### Results

#### Response Times

**FIGURE 5:**
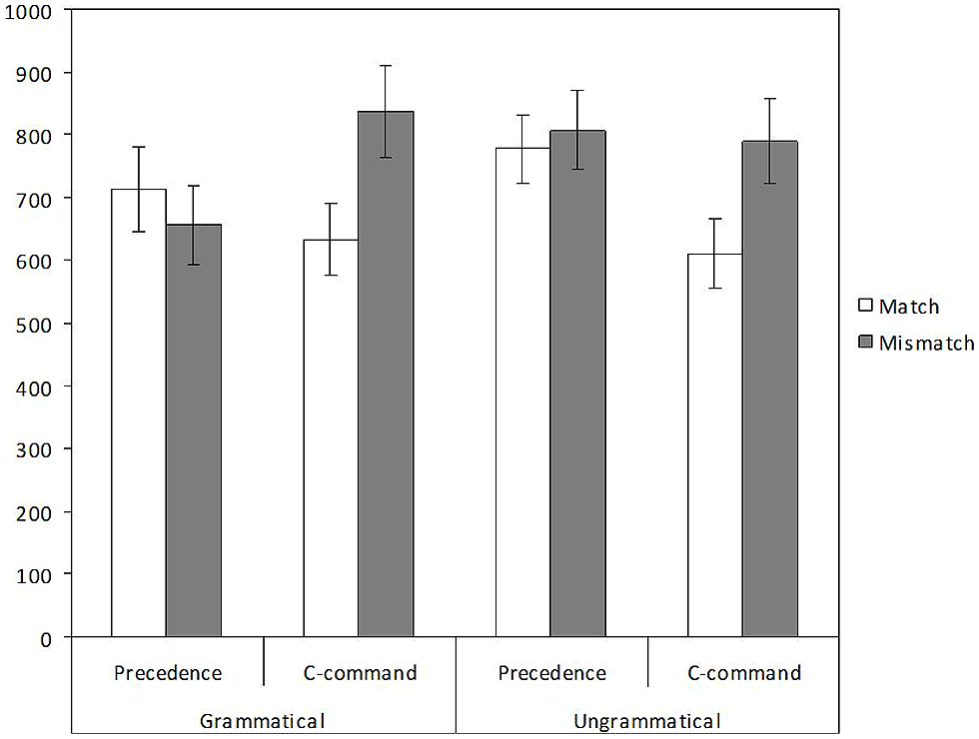
Distribution of RTs (ms) for correct judgments in the speeded grammaticality judgment of Experiment 3

The distribution of response times is illustrated in **Figure 5**. The model showed a main effect of number (β = -100.73, *t* = -2.213, *p* = 0.027), with slower response times in the condition with plural objects (766 ms) as compared to the condition with singular objects (658 ms). There was no effect of structure, and no effect of grammaticality (*ts* < 1). The predicted interaction between number and structure was significant (β = 212.50, *t* = 2.341, *p* = 0.019), and failed to interact with grammaticality, as attested by the non-significant three-way interaction (*t* < 1). Models run separately on each structure showed a significant effect of number of the c-commanding element (β = -213.31, *t* = -3.173, *p* = 0.002), with slower RTs for plural c-commanding elements (790 ms) than for singular ones (619 ms), but no significant effect of number of the preceding element (742 vs. 696 ms, *t* < 1). Grammaticality played no role in these models (*ts* < 1).

#### Accuracy

**FIGURE 6:**
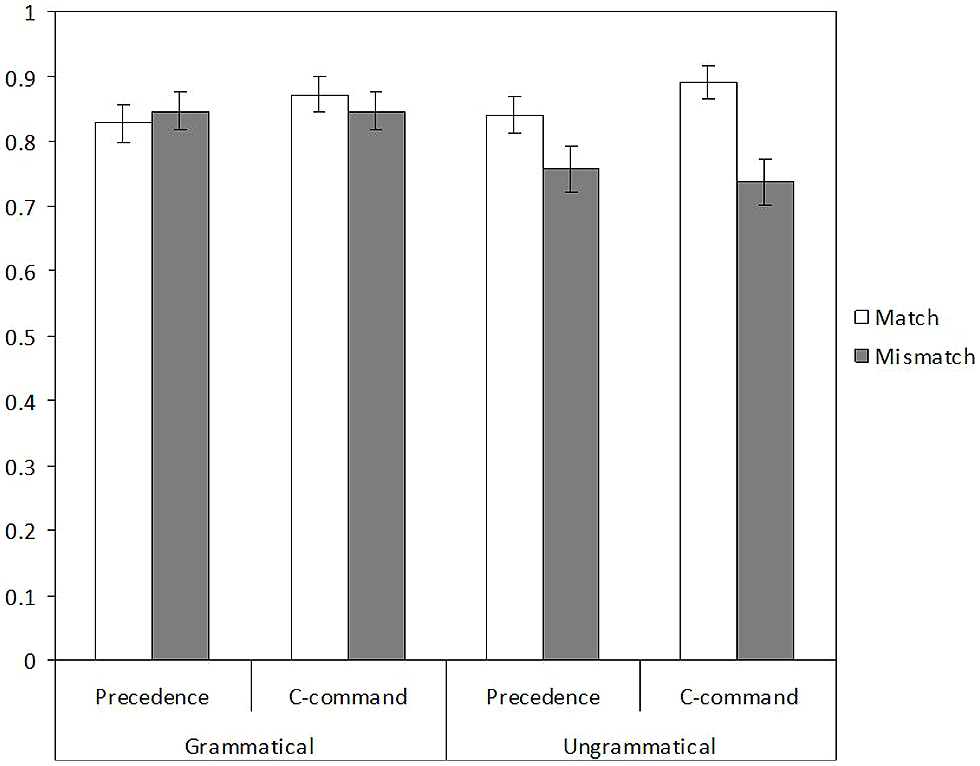
Accuracy proportions in the speeded grammaticality judgment of Experiment 3

Mean accuracy scores are reported in **Figure 6**. The model showed a main effect of number (β = 0.442, *z* = 2.77, *p* = 0.006), with lower accuracy rates with plural attractors (0.80) than with singular ones (0.86), as well as an interaction between number and grammaticality (β = 0.817, *z* = 2.567, *p* = 0.010). The interaction between number and structure failed to reach significance level (β = -0.471, *z* = -1.48, *p* = 0.138). Splitting the interaction into two separate models showed that whereas number significantly affected accuracy in ungrammatical sentences (β = 0.817, *z* = 3.926, *p* < 0.001), with better scores in the number match condition (0.87) than in the mismatch condition (0.75), it did not affect it in grammatical sentences (*z* < 1). Number and structure failed to interact significantly in the two models (*z* < 1).

### Discussion

Experiment 3 brings two new findings. First, we found an effect of the structural variable manipulated: whereas the plural mismatching feature on the DP intervening in terms of c-command on agreement significantly contributed to slowing grammaticality judgments as compared to the singular matching feature, the plural feature on the DP intervening in terms of precedence failed to significantly affect response times. The finding that c-command intervention creates stronger interference than precedence intervention replicates previous reports in sentence production, with yet different constructions. Data from sentence production showed that the accusative clitic object pronoun situated pre-verbally creates more interference than a PP modifier situated in the same linear position (Franck et al., 2006) and more interference than the preverbal dative clitic (Franck et al., 2010). Both the PP modifier and the dative can be argued to intervene on the agreement dependency in terms of precedence, being embedded in a prepositional layer, whereas the accusative clitic intervenes in terms of c-command.

Second, grammaticality does not impact on performance in a speeded grammaticality judgment task: the same interference effect is found independently of whether the sentence is grammatical or not (see Häussler, unpublished, for a similar finding in German). This finding suggests that the differences in the direction of the number interference effect between Experiment 1 (self-paced reading), showing similarity-based interference, and Experiment 2 (speeded grammaticality judgment), showing attraction, is not due to the fact that that the former tested grammatical sentences whereas the latter tested ungrammatical sentences. Indeed, Experiment 3 shows that interference always shows up as an attraction effect in speeded grammaticality judgment. Hence, what seems critical is the process that the task taps into: whereas self-paced reading taps into the process of structure building and resolution of an A’-dependency, facilitated by the presence of a number mismatching feature on the subject intervening on the object-gap dependency, grammaticality judgment taps into the process of agreement checking, penalized by the presence of a number mismatching feature on the object intervening on the subject-verb dependency.

Finally, response times in Experiment 3 are faster than in Experiment 2, which showed particularly slow responses. The two experiments also differ in their overall error rates: in Experiment 2 where response times were between 1 and 2 s, the error rate was smaller than 10% overall; in contrast, the error rate in Experiment 3 where response times were between 600 and 850 ms was between 15 and 25%. It may therefore be the case that participants granted a privileged status to accuracy in Experiment 2. Nevertheless, it is important to note that there was no trade-off between response times and error rates across conditions in Experiment 3: conditions that were slower were also those that generated more errors.

## General Discussion

We reported three studies exploring the consequences of a number featural mismatch in the comprehension of structures involving intervention configurations. The structures manipulated shared similar superficial characteristics, but critically differed in their hierarchical configurations. Experiments 1 and 2 contrasted object relatives clauses, involving an intermediate position created by movement of the object and intervening on the subject-verb agreement relation, and sentence complement clauses in which the object fails to intervene on the agreement relation at any point in the derivation of the hierarchical structure. Experiment 3 contrasted two structures involving complex objects also intervening on agreement in the object’s intermediate position, but differing in the hierarchical position of the number mismatching feature situated either in a position of intervention in terms of c-command on agreement, or in terms of linear precedence.

The comparison between the first two experiments conducted on the same materials shows that self-paced reading (Experiment 1) and grammaticality judgment (Experiment 2) tap into distinct processes differently sensitive to intervention. The combination of the last two grammaticality judgment experiments illustrates the role of fine aspects of the hierarchical structure in agreement processing in sentence comprehension. Task-dependency and structure-dependency of number interference effects are discussed in turn.

### Task-Dependent Interference in the Process of Structure Building

Experiment 1 using a self-paced reading procedure with grammatical sentences showed that participants read the verb significantly faster in the presence of a mismatching plural object in the relative clause, while no effect of number was found in the complement clause. Experiment 2 using a grammaticality judgment task specifically focusing on subject-verb agreement with the use of ungrammatical sentences also found an effect of the object’s number restricted to relatives. However, this effect was reversed, with slower grammaticality judgments in the presence of a plural object, in line with attraction effects found in sentence production (Bock and Miller, 1991; Franck et al., 2006, 2010).

What is the mechanism underlying interference in the two experiments? We have suggested that both experiments reflect intervention effects on the hierarchical structure; however, the two experiments, because of the different techniques used, tap into two distinct processes, highlighting two different kinds of intervention effects. Experiment 1 reflects subject intervention on the object A’-dependency, Experiment 2 reflects object intervention on the subject-verb agreement dependency. More particularly, we have argued that self-paced reading taps into the process of structure building, in which the parser needs to resolve the required antecedent-gap dependency and assign the appropriate theta-roles to the arguments of the verb. The data of Experiment 1 are in line with recent developmental research attesting to children’s better understanding of object relatives when the subject and object mismatch in number (Adani et al., 2010; Adani, unpublished). Similarly, mismatches in other features have also been found to facilitate object relative clause comprehension: gender mismatch (Belletti et al., 2012), animacy mismatch (e.g., Mak et al., 2002, 2006; Traxler et al., 2002) or mismatch in the NP-type (DP, pronoun, proper name; e.g., Gordon et al., 2001, 2004; Warren and Gibson, 2002, 2005; Grillo, 2009; Belletti and Rizzi, 2013). Capitalizing on the theory of Relativized Minimality (Rizzi, 1990, 2004), Friedmann et al. (2009) suggested that the difficulty in building A’-dependencies in object relatives stems from the intervention of the subject DP in the path connecting the relative head and its trace in object position. Critically, the difficulty is hypothesized to be a function of the degree of overlap in syntactic features between the relative head and the intervener. According to this set-theoretic approach, the minimal degree of distinctness, identity, excludes the configuration from the grammar, while the maximal degree of distinctness, disjunction, makes the configuration fully accessible to both children and adults. The intermediate cases of inclusion and intersection would respectively engender stronger and weaker difficulty, the former manifesting itself in terms of the failure to build the A’-dependency in children and of a significant slowing down of processing in adults. In this framework, the facilitating role of number mismatch is captured in terms of the set theoretic relation in featural specification of the intervener with respect to the target.

The approach we have assumed expresses the intervention effect and the amelioration observed with feature mismatch directly in terms of a grammatical constraint, Relativized Minimality. Alternative approaches rooted in the psycholinguistic tradition do not appeal to a particular grammatical constraint and directly focus on the process of retrieving the object from memory when the verb is reached in parsing. Memory retrieval models assume that retrieval in long-distance dependencies involves a cue-based mechanism operating on content-addressable memory representations (e.g., McElree et al., 2003; Van Dyke and Lewis, 2003; Lewis and Vasishth, 2005; Van Dyke and McElree, 2006). These models grant a key role to similarity-based interference, which arises when memory units other than the retrieval target partially overlap with it in terms of their syntactic or semantic make-up. Although these models capture various interference effects reported in the literature (e.g., Lewis et al., 2006) only few attempts have tried to understand how the memory mechanisms posited can capture complex relational syntactic constraints (e.g., Vasishth et al., 2008; Dillon et al., 2013; Alcocer and Phillips, unpublished). One possible way in which our interpretation based on Relativized Minimality and more standard psycholinguistic approaches in terms of cue-based object retrieval may differ concerns the locus of the interference effect. If our interpretation is on the right track, the observed faster reading in the mismatch condition in sentences like (1a) has no direct relation with subject-verb agreement on the verb: it simply has to do with the resolution of an (object) A’-dependency across a partially matching intervener (the subject). If this is correct, we would expect the mismatch effect to enhance reading times at the verb (when the object trace is postulated and the A’-dependency resolved) even if the verb is uninflected, as for example in a sentence with a modal in English (e.g., *John talked to the patient(s) that the medicine can cure*), or in a sentence with an infinitival verb. If, as assumed in cue-based retrieval models, number on the verb serves as a linking address to memory units, the number effect should disappear with uninflected verbs. We intend to test this prediction in future work.

If a cue-based retrieval mechanism is at play in Experiment 1, it is, in any case, of a different type from the one assumed by Wagers et al. (2009) who tied it to a process of agreement ‘rechecking,’ triggered by the unpredicted number feature on the erroneously agreed verb. The number effect in Experiment 1 was found on grammatical sentences and in the verb region, while Wagers et al. (2009) found it in ungrammatical sentences and in the post-verbal region. These differences in the data suggest that if memory retrieval is responsible for the effect here, it must be tied to an early process of structure building and not to a late process of rechecking after the structure has been built, as proposed by Wagers et al. (2009). One could then wonder why Wagers et al. (2009) failed to find number interference in grammatical sentences in their work. The two studies differ in at least two respects. First, whereas Wagers et al. (2009) tested both grammatical and ungrammatical sentences, our materials only involved grammatical sentences. The presence of agreement errors in the English materials may have contributed to artificially disqualify number as a relevant cue to parsing, therefore explaining the lack of an effect in grammatical sentences. Second, our materials involved a mix of superficially similar object relatives and complement clauses; one cannot exclude the possibility that having to switch from one structure to the other increased the processing burden on structure building. The two factors may have played a cumulative role in the differences observed between the two studies.

The finding that the number effect in object relatives was reversed when measured in the grammaticality judgment task of Experiment 2, and turned into an attraction effect similar to the one found in sentence production, was taken as evidence that the task tapped into a different process. The grammaticality judgment task indeed forces the parser to first build the hierarchical structure over which agreement can be calculated, and can therefore be reasonably thought of as tapping into agreement computation proper. The finding that number interference arises as an attraction effect, and that the effect is restricted to object relatives and fails to manifest in complement clauses, suggests that the same mechanism underlies agreement computation in comprehension and production. In the next section, we describe our view of that mechanism.

### Structure-Dependent Attraction in Agreement in Sentence Comprehension

In contrast to Experiment 1, Experiments 2 and 3 both showed that the presence of a number mismatching feature in the sentence significantly penalizes grammaticality judgments. Although Experiment 2 only tested ungrammatical sentences, Experiment 3 showed that the number effect arose independently of whether the sentence is grammatical or ungrammatical.

Results of Experiment 2 showed that attraction arises only in object relatives, when the attractor is the moved object of the target verb in the relative, but not when it is the object of the main verb in sentence complements, despite the superficial similarity of the two structures. Experiment 3 showed that a mismatching feature in a moved complex object intervening by c-command on an agreement configuration generates more attraction than one intervening by precedence. These two findings replicate our previous reports in sentence production (Franck et al., 2006, 2010), arguing in favor of identical syntactic representations over which agreement takes place in production and comprehension (e.g., Nicol et al., 1997; Pearlmutter et al., 1999; Thornton and MacDonald, 2003; Hartsuiker, 2006; Badecker and Kuminiak, 2007).

What are the operating principles of agreement computation? In our production research, we suggested that attraction arises because of the intervention, on the subject-verb (AGREE) dependency, of the object transiting in its intermediate position at the periphery of the vP (Franck et al., 2006, 2010). Intervention by the intermediate object trace created by object movement, was argued to set the necessary condition for interference to arise. On that view, attraction results from the incorrect feature passing from the object to the verb via AGREE. One could nevertheless entertain a different scenario to account for the report of attraction in object relatives but not in sentence complements. A vast literature suggests that the parser reactivates the moved object when reaching the verb that it is an argument of (e.g., Stowe, 1986; McElree et al., 2003; Fedorenko et al., 2013). Hence, one cannot exclude the possibility that interference arises because the object is active during the same time window as the subject. Against this hypothesis, experimental evidence from sentence production shows that attraction arises even for moved objects that are not arguments of the target verb, as in (4).

(4) Voici les otages que le journaliste ^∗^apprennent qu’on a blessés. *Here are the hostages-PL that the journalist-SG ^∗^learn-PL that someone injured.*

Moreover, the strength of the attraction effect in this context is identical to attraction from the verbal argument tested in the context of object relatives (*John speaks to the patients that the medicine ^∗^cure*; Franck et al., 2010, Experiment 4). Hence, in order for a blind memory reactivation account to capture this report, one would need to assume that the parser reactivates all noun phrases from the parsed tree at the target verb to the same extent: *hostages* in (4) should be reactivated at the verb *learn*, of which it is not an argument, to a similar extent as the argument *patients* is reactivated at *cure* in the object relative clause. Even though retrieval is indeed known to be sensitive to interference from non-target elements sharing cues with the target (e.g., Van Dyke and McElree, 2006), it is marginal since, in the vast majority of the cases, the correct target is retrieved. Thus, a simple memory activation model fails to capture our finding that a moved object that is not part of the argument structure of the target verb triggers similar attraction to a moved verbal argument. The critical explanatory factor in interference rather appears to be the intervention of a moved DP in the hierarchical subject-verb dependency, a configuration which is identical whether the moved DP is an argument of the critical verb or not.

Results of Experiment 3 bring further support to our previous finding in agreement production that c-commanding interveners are more prone to trigger attraction than preceding ones. C-command has played a key role in syntactic theory, ever since work by Reinhart (unpublished), and has pervasive consequences on various morphosyntactic and interpretive processes like the binding of anaphors and the proper scope interpretation of quantifiers. The AGREE operation by which the subject’s features are copied onto the agreement node in the functional layer of the clause also takes place under the constraint of c-command. Experiment 3 shows that an intervening object trace disrupts the processing of subject-verb agreement when a mismatching number feature intervenes between the subject and the verbal inflection in the hierarchical terms of c-command, and does so significantly more than when it intervenes in terms of mere precedence. This result parallels previous results on the stronger interference triggered by a c-commanding intervener in sentence production (Franck et al., 2010). In conclusion, both production and comprehension systems show a parallel sensitivity to the hierarchical relation of c-command, which thus has a central role both in grammar and performance.

Tanner et al. (2014) proposed that maintaining a unified account of agreement in production and comprehension minimally requires (1) that the same factors that modulate attraction in production also modulate attraction in comprehension, and (2) that interference in comprehension is symmetrical, as in production, meaning that attraction is expected to manifest independently of whether the sentence is grammatical or not in comprehension, or whether the correct verb form is ultimately chosen or not in production (a slowing down has been observed in the presence of a plural attractor in production even if correct agreement was used on the verb, Staub, 2009, 2010; Brehm and Bock, 2013). Results from the speeded grammaticality judgment Experiments 2 and 3 meet these requirements, suggesting that even though number interference effects may arise from different causes depending on the task used to measure sentence comprehension, the mechanism of agreement computation itself is the same in production and comprehension. This mechanism appears to operate under fine constraints as defined in formal syntax, including movement, intermediate traces and c-command.

## Conclusion

The finding that the same structural effects as those found in sentence production are found in sentence comprehension is relevant both at the theoretical level and at the methodological levels. At the theoretical level, it argues in favor of a common syntactic component shared by production and comprehension, in spite of the obvious differences due to the intrinsically anticipatory nature of the parser. We suggested that the common component of agreement shows up when the comprehension task allows it to, as is the case when participants are required to judge the grammaticality of the sentence under time constraints. At the methodological level, grammaticality judgment is much easier to use than elicitation tasks, which often produce a very small range of errors that are problematic to analyze statistically. Speeded grammaticality judgment allows measuring not only errors but also response times, hence providing a finer measure necessary for subtle syntactic variables to show up in an otherwise noisy performance. It therefore offers an ideal tool for the future exploration of the core syntactic components of agreement computation.

## Acknowledgments

This work was supported by grant 100014-126924 from the Swiss National Fund for Scientific Research to Julie Franck. We wish to thank Brian Dillon, Akira Omaki, Whit Tabor and Matt Wagers for enriching discussions, and Maria Lucia Calí for data collection. We take complete responsibility for the content of the paper.

## Supplementary Material

The Supplementary Material for this article can be found online at: http://www.frontiersin.org/journal/10.3389/fpsyg.2015.00349/abstract
