# Corrigendum: Task-dependency and structure-dependency in number interference effects in sentence comprehension

**DOI:** 10.3389/fpsyg.2015.00807

**Published:** 2015-06-09

**Authors:** Julie Franck, Saveria Colonna, Luigi Rizzi

**Affiliations:** ^1^Laboratoire de Psycholinguistique, University of GenevaGeneva, Switzerland; ^2^Centre National de la Recherche Scientifique – University of Paris 8Paris, France; ^3^Department of Linguistics, University of GenevaGeneva, Switzerland; ^4^Interdepartmental Centre for Cognitive Studies of Language, University of SienaSiena, Italy

**Keywords:** number, agreement, attraction, intervention, intermediate traces, c-command, cue-based retrieval, comprehension

The reference of the following sentence should be Adani et al. (2014) rather than Adani (unpublished).

“Adani et al. (2010) and Adani (unpublished) found that both English and Italian speaking children showed better performance in a sentence-picture matching task when the object and the subject of the object relative clause mismatched in number (e.g., Show me the elephant that the lions are washing is better understood than Show me the lion that the elephant is washing).”

Adani et al. (2014) should thus be added to the References.

## Conflict of interest statement

The authors declare that the research was conducted in the absence of any commercial or financial relationships that could be construed as a potential conflict of interest.
