# Supplementary material for: Task-dependency and structure-dependency in number interference effects in sentence comprehension
Source: Front Psychol. 2015 Apr 10;6:349. doi: 10.3389/fpsyg.2015.00349 (PMC4392591; doi:10.3389/fpsyg.2015.00349)
Supplement: Supplementary file 1 [file Table_1.DOCX]

***Supplementary material***

**Task-dependency and structure-dependency in number interference effects in sentence comprehension**

Julie Franck^1^, Saveria Colonna^2^, Luigi Rizzi^3^

^1^University of Geneva

^2^University of Paris 8 and CNRS

^3^University of Geneva and University of Siena

**Correspondence**: Dr. Julie Franck, Laboratoire de Psycholinguistique, Université de Genève, 40 Boulevard du Pont d'Arve, 1205 Geneva, Switzerland.

[Julie.Franck@unige.ch](mailto:Julie.Franck@unige.ch)

**1. Supplementary Items**

*Items used in Experiments 1 and 2, in the mismatch condition. Note that in Experiment 2, the last two windows were missing such that the sentence ended with the verb. SC = Sentence Complement ; RC = Relative clause*

SC: Jules / signale / aux étudiants / que / l'orateur / bâille / sérieusement / depuis le début.

RC: Jules / sourit / aux étudiants / que / l'orateur / endort / sérieusement / depuis le début.

SC: Paul / apprend / aux électeurs / que / le politicien / ment / franchement / depuis les élections.

RC: Paul / pense / aux électeurs / que / le politicien / déçoit / franchement / depuis les élections.

SC: Sébastien / avoue / aux patientes / que / le calmant / agit / progressivement / depuis un an.

RC: Sébastien / sourit / aux patientes / que / le calmant / affaiblit / progressivement / depuis un an.

SC: Jérôme / signale / aux prisonnières / que / le gardien / vient / parfois / dans la cour.

RC: Jérôme / parle / aux prisonnières / que / le gardien / sort / parfois / dans la cour.

SC: Charles / explique / aux victimes / que / le traitement / démarre / lentement / mais sûrement.

RC: Charles / sourit / aux victimes / que / le traitement / guérit / lentement / mais sûrement.

SC: Benjamin / rappelle / aux adolescents / que / l'éducateur / boit / souvent / depuis quelques années.

RC: Benjamin / pense / aux adolescents / que / l'éducateur / bat / souvent / depuis quelques années.

SC: Bernard / rappelle / aux joueuses / que / le casino / ferme / malheureusement / bien trop vite.

RC: Bernard / songe / aux joueuses / que / le casino / ruine / malheureusement / bien trop vite.

SC: Laure / dit / aux bergères / que / le mouton / bêle / stupidement / après la tonte.

RC: Laure / sourit / aux bergères / que / le mouton / suit / stupidement / après la tonte.

SC: Pierre / annonce / aux candidates / que / le jury / délibère / fermement / après l'audition.

RC: Pierre / téléphone / aux candidates / que / le jury / attend / fermement / après l'audition.

SC: Marc / répète / aux populations / que / le malheur / perdure / fatalement / après le drame.

RC: Marc / pense / aux populations / que / le malheur / unit / fatalement / après le drame.

SC: Claire / rappelle / aux ouvriers / que / la cheminée / fume / énormément / depuis les travaux.

RC: Claire / plaît / aux ouvriers / que / la cheminée / noircit / énormément / depuis les travaux.

SC: Patricia / dit / aux clientes / que / le chapeau / plaît / beaucoup / à cause des plumes.

RC: Patricia / parle / aux clientes / que / le chapeau / grandit / beaucoup / à cause des plumes.

SC: Fred / raconte / aux enfants / que / le curé / part / joyeusement / après chaque messe.

RC: Fred / sourit / aux enfants / que / le curé / bénit / joyeusement / après chaque messe.

SC: Lise / promet / aux femmes / que / le régime / réussit / étonnamment / facilement.

RC: Lise / parle / aux femmes / que / le régime / amincit / étonnamment / facilement.

SC: Gilles / rappelle / aux travailleuses / que / l'effort / redouble / inexorablement / avec le temps.

RC: Gilles / parle / aux travailleuses / que / l'effort / fatigue / inexorablement / avec le temps.

SC: Jacques / explique / aux patronnes / que / le stress / arrive / prématurément / malgré les anxiolytiques.

RC: Jacques / pense / aux patronnes / que / le stress / vieillit / prématurément / malgré les anxiolytiques.

SC: Patrick / répète / aux familles / que / la fête / dure / chaque année / pendant dix jours.

RC: Patrick / pense / aux familles / que / la fête / réunit / chaque année / pendant dix jours.

SC: Louise / répète / aux gamines / que / la sorcière / grimace / volontairement / pour Halloween.

RC: Louise / sourit / aux gamines / que / la sorcière / inquiète / volontairement / pour Halloween.

SC: Aude / répète / aux gymnastes / que / le massage / débute / toujours / après l'entraînement.

RC: Aude / plaît / aux gymnastes / que / le massage / détend / toujours / après l'entraînement.

SC: Luc / dit / aux fillettes / que / le dragueur / bavarde / inlassablement / depuis une heure.

RC: Luc / pense / aux fillettes / que / le dragueur / séduit / inlassablement / depuis une heure.

SC: Anne / rappelle / aux actrices / que / le spectateur / rigole / frénétiquement / à chaque représentation.

RC: Anne / parle / aux actrices / que / le spectateur / applaudit / frénétiquement / à chaque représentation.

SC: Jeanne / rappelle / aux voisines / que / la sortie / échoue / rarement / en fin d'année.

RC: Jeanne / parle / aux voisines / que / la sortie / excite / rarement / en fin d'année.

SC: Jean / répète / aux avocats / que / la déception / persiste / inévitablement / après le procès.

RC: Jean / téléphone / aux avocats / que / la déception / aigrit / inévitablement / après le procès.

SC: Roland / indique / aux canailles / que / le policier / intervient / secrètement / depuis un mois.

RC: Roland / sourit / aux canailles / que / le policier / poursuit / secrètement / depuis un mois.

*Items used in Experiment 3, in the mismatch condition. The first sentence is the c-command condition, the second the precedence condition.*

Quelles patientes du médecin dis-tu que le juriste défend/*défendent ?

Le chirurgien de quelles patientes dis-tu que le juriste défend/*défendent ?

Quelles secrétaires du ministre dis-tu que le syndicaliste contacte/*contactent ?

L'adjoint de quelles secrétaires dis-tu que le syndicaliste contacte/*contactent ?

Quels ingénieurs du constructeur dis-tu que le concurrent corrompt/*corrompent ?

Le superviseur de quels ingénieurs dis-tu que le concurrent corrompt/*corrompent ?

Quelles nageuses de l'équipe dis-tu que l'arbitre interrompt/*interrompent ?

L’entraîneur de quelles nageuses dis-tu que l'arbitre interrompt/*interrompent ?

Quelles animatrices de l'enfant dis-tu que le parent menace/*menacent ?

L’aide de quelles animatrices dis-tu que le parent menace/*menacent ?

Quelles chanteuses du groupe dis-tu que le public estime/*estiment ?

Le pianiste de quelles chanteuses dis-tu que le public estime/*estiment ?

Quels experts du peintre dis-tu que l'acheteur irrite/*irritent ?

Le consultant de quels experts dis-tu que l'acheteur irrite/*irritent ?

Quels ouvriers du contremaître dis-tu que l'architecte questionne/*questionnent ?

Le contremaître de quels ouvriers dis-tu que l'architecte questionne/*questionnent ?

Quels gardes du corps du ministre dis-tu que l'enquêteur alerte/*alertent ?

Le chef de quels gardes du corps dis-tu que l'enquêteur alerte/*alertent ?

Quels caméramans du stagiaire dis-tu que l'acteur énerve/*énervent ?

Le stagiaire de quels caméramans dis-tu que l'acteur énerve/*énervent ?

Quels viticulteurs de l’apprenti dis-tu que le sommelier rencontre/*rencontrent ?

L'apprenti de quels viticulteurs dis-tu que le sommelier rencontre/*rencontrent ?

Quels traducteurs du diplomate dis-tu que le président félicite/*félicitent ?

L’associé de quels traducteurs dis-tu que le président félicite/*félicitent ?

Quels livreurs du pâtissier dis-tu que le douanier contrôle/*contrôlent ?

L’ami de quels livreurs dis-tu que le douanier contrôle/*contrôlent ?

Quels banquiers du patron dis-tu que le commissaire traque/*traquent ?

L'actionnaire de quels banquiers dis-tu que le commissaire traque/*traquent ?

Quelles stylistes du mannequin dis-tu que le créateur reçoit/*reçoivent ?

Le modèle de quelles stylistes dis-tu que le créateur reçoit/*reçoivent ?

Quels relieurs de l'éditeur dis-tu que le publiciste conteste/*contestent ?

Le responsable de quels relieurs dis-tu que le publiciste conteste/*contestent ?

Quelles formatrices du bachelier dis-tu que le directeur interroge/*interrogent ?

L’élève de quelles formatrices dis-tu que le directeur interroge/*interrogent ?

Quels comptables du notaire dis-tu que la police soupçonne/*soupçonnent ?

Le remplaçant de quels comptables dis-tu que la police soupçonne/*soupçonnent ?

Quels assistants du professeur dis-tu que le délégué tutoie/*tutoient ?

Le responsable de quels assistants dis-tu que le délégué tutoie/*tutoient ?

Quels cuisiniers de l'hôtelier dis-tu que le milliardaire engage/*engagent ?

Le commis de quels cuisiniers dis-tu que le milliardaire engage/*engagent ?

Quels secouristes de la blessée dis-tu que le pompier remercie/*remercient ?

Le formateur de quels secouristes dis-tu que le pompier remercie/*remercient ?

Quels thérapeutes du patient dis-tu que l'assureur rembourse/*remboursent ?

Le patient de quels thérapeutes dis-tu que l'assureur rembourse/*remboursent ?

Quelles touristes du club dis-tu que l'organisateur conseille/*conseillent ?

Le chauffeur de quelles touristes dis-tu que l'organisateur conseille/*conseillent ?

Quels soldats du lieutenant dis-tu que l'espion surveille/*surveillent ?

L’officier de quels soldats dis-tu que l'espion surveille/*surveillent ?

**2. Supplementary Analyses**

The distribution of response times for the incorrect grammaticality judgments in Experiment 3 is reported in the table below:

|  |  | Grammatical | Ungrammatical |  |
| --- | --- | --- | --- | --- |
|  | Precedence | C-command | Precedence | C-command |
| Match | 452 | 297 | 440 | 967 |
| Mismatch | 709 | 638 | 857 | 732 |

The model involving accuracy (correct vs. incorrect response) shows that the number*structure interaction, significant in the analysis of correct responses, interacts with accuracy here, in that it shows an opposite profile:

Fixed effects:

|  | Estimate | Std. Error | df | t value | Pr(>\|t\|) |
| --- | --- | --- | --- | --- | --- |
| (Intercept) | 681.78 | 78.99 | 68.10 | 8.631 | 1.55e-12 *** |
| nb1 | -136.66 | 103.17 | 1216.30 | -1.325 | 0.1855 |
| struc1 | -32.66 | 103.66 | 346.50 | -0.315 | 0.7529 |
| acc | 38.28 | 58.02 | 1232.30 | 0.660 | 0.5095 |
| nb1:struc1 | -337.28 | 204.20 | 1200.10 | -1.652 | 0.0989 |
| nb1:acc | 36.20 | 113.24 | 1218.70 | 0.320 | 0.7492 |
| struc1:acc | 47.74 | 112.87 | 1019.60 | 0.423 | 0.6724 |
| nb1:struc1:acc | 558.74 | 223.92 | 1196.40 | 2.495 | 0.0127 |

The model run on incorrect responses only shows a marginal interaction between number and structure, in the opposite direction from the interaction found on correct responses: the increase in RTs in the presence of a plural attractor as compared to a singular one is stronger in the condition of precedence intervention than in the condition of c-command intervention:

Fixed effects:

| E | Estimate | Std. Error | df | t value | Pr(>\|t\|) |
| --- | --- | --- | --- | --- | --- |
| (Intercept) | 713.89 | 89.07 | 21.58 | 8.015 | 6.58e-08 *** |
| gram1 | 158.12 | 107.27 | 200.16 | 1.474 | 0.1420 |
| nb1 | -128.95 | 106.98 | 197.49 | -1.205 | 0.2295 |
| struc1 | -64.42 | 105.56 | 178.29 | -0.610 | 0.5424 |
| gram1:nb1 | 130.91 | 209.18 | 191.75 | 0.626 | 0.5322 |
| gram1:struc1 | -209.95 | 209.18 | 190.03 | -1.004 | 0.3168 |
| nb1:struc1 | -357.70 | 209.96 | 192.93 | -1.704 | 0.0901 |
| gram1:nb1:struc1 | -412.27 | 432.06 | 203.63 | -0.954 | 0.3411 |

The global picture that seems to emerge is that the condition that showed slower response times on accurate responses (plural, c-commanding intervener) shows faster response times on erroneous responses, that is, a reversed profile. In other words, the data on incorrect responses are in line with the finding of a structural modulation of attraction in correct responses: participants are faster to get agreement wrong (i.e., be lured and show an illusion of (un)grammaticality) in the presence of a c-commanding plural intervener than in the presence of a preceding plural intervener.
